# Initial experience with off-pump left ventricular assist device implantation in single center: retrospective analysis

**DOI:** 10.1186/1749-8090-5-123

**Published:** 2010-12-06

**Authors:** Hamdy Awad, Mohamed Abd El Dayem, Jarrett Heard, Galina Dimitrova, Lianbo Yu, Benjamin C Sun

**Affiliations:** 1 The Ohio State University Medical Center, Department of Anesthesiology, N411 Doan Hall, 410 West 10th Avenue, Columbus, OH, 43210, USA; 2 The Ohio State University College of Medicine, 370 West 9th Avenue, Columbus, OH, 43210, USA; 3 The Ohio State University Medical Center, Center for Biostatistics, 2012 Kenny Road, Columbus, OH, 43221, USA; 4 The Ohio State University Medical Center, Department of Surgery, Division of Cardiothoracic Surgery, N847 Doan Hall, 410 West 10th Avenue, Columbus, OH, 43210, USA

## Abstract

**Background:**

We hypothesize that implantation of left ventricular assist device through off-pump technique is feasible and has a comparable result to implantation on cardiopulmonary bypass and could improve one-year survival.

**Methods:**

This retrospective, observational, single-center study was conducted on 29 consecutive patients at our institution who underwent off-pump left ventricular assist device implantation by a single surgeon.

**Results:**

Twenty-seven procedures were performed successfully using the off-pump technique. The survival rate was 92% at 30 days, 76% at 90 days, and 67% at one year. We compared the one-year survival of different implantation periods, and divided our study into three time intervals (2004-2005, 2006, and 2007). There was a trend in reduction in number of deaths over one year that demonstrated a decrease in death rate from 50% to 17%, as well as improvement in our experience over time. However, this trend is not statistically significant (p = 0.08) due to limited sample size.

**Conclusions:**

Based upon our findings, off-pump left ventricular assist device implantation is a feasible surgical technique, and combining this technique with improved device technology in the future may provide even greater improvement in patient outcomes.

## Background

Left ventricular assist devices were approved as a bridge to transplantation therapy (BTT) in 1998 [1]. In 2001, they became a destination therapy in the United States [2]. Perioperative complications result from a combination of three factors: 1) intrinsic heart failure with secondary organ damage; 2) long-term effect of the implanted device; and 3) surgical techniques employed, including cardiopulmonary bypass (CPB). CPB has advantages such as inspecting left ventricle for thrombus, hemodynamic resuscitation, and removing fluid, including ultrafiltrations on CPB, but it may have unwanted effects as well, such as increasing systemic inflammatory response and transfusion requirements. To date, the experimental and clinical data comparing on-pump and off-pump coronary surgery suggest an affected cardiac function in favor of off-pump operations, which might be related to greater myocardial damage during hypothermic CPB operations [3]. We believe in order to improve the clinical outcome there is a need to ameliorate one of these factors. Although previous studies have demonstrated an advantage in reducing morbidity in off-pump coronary artery bypass-graft (CABG) patients, but not reducing mortality, it can be reasonably concluded that the same may hold true in sicker patients receiving off-pump left ventricular assist device (OP LVAD) implantation in high-risk heart failure patients [4,5]. This article analyzes our experience with the initial series of 29 consecutive patients undergoing placement of LVAD as both BTT and destination therapy, without the use of CPB, and measures postoperative mortality up to one year.

## Methods

After obtaining approval from the Institutional Review Board, we retrospectively reviewed data from patients who were scheduled to undergo OP LVAD implantation at The Ohio State University Medical Center between October 2004 and May 2007. Postoperative outcomes included ventilation hours and postoperative hospital stays (days). Mean and standard deviation were calculated [5]. Early postoperative bleeding was in accordance with Interagency Registry for Mechanically Assisted Circulatory Support (INTERMACS) guidelines to ensure that the descriptions of adverse events in studies using mechanical assist devices are universal. The types of implanted devices included: 14 HeartMate II, 10 HeartMate XVE, 2 IVAD, and 1 DeBakey. The incidence rates of complications were calculated. Patient mortality was calculated at three time periods: 30 days, 90 days, and one year postoperatively. We performed a univariate analysis of all patient data to obtain descriptive statistics. Comparisons across categories were performed through Fisher's exact test.

### Anesthetic Technique

We reviewed the intraoperative anesthetic chart in all study patients, which included the monitoring of arterial blood pressure, heart rate, pulmonary artery pressure, and central venous pressure. An intraoperative transesophageal echocardiogram was employed for assessment of right and left ventricular function, exclusion of associated pathologies that preclude OP implantation (intra-atrial shunts, ventricular thrombus, severe aortic insufficiency), and guidance in de-airing of the heart once the device was implanted. Heparin was administered to keep the kaolin activated clotting time (ACT) above 300 seconds. Inotropic medications were used to support right ventricular function according to the treating anesthesiologist. There was no point of care testing for transfusion requirements, which were determined by the treating physicians. The treating physician made clinical decisions about the transfusion of PRBCs or other blood products without clear logarithms.

### Surgical Technique

A detailed surgical technique of OP LVAD implantation was first perfected after more than 30 intracorporeal ventricular assist devices were implanted off pump in bovine and ovine models for various studies including heart failure (unpublished data) due to the time- and cost-intensive nature of placing animals on CPB [6]. Our team's experience with this novel idea was then translated to our patients. Before starting the procedure, we performed transesophageal echocardiography to monitor underlying contraindications to OP LVAD implantation, such as severe aortic regurgitation, right-to-left shunts with worsening hypoxemia, and/or thrombus formation. Also, a primed bypass circuit was available in case of an emergent change to an on-pump procedure, such as hemodynamic instability. After median sternotomy, a pericardial well was created and a pre-peritoneal pocket formed by tunneling the driveline through the skin, with placement of the device in the pocket. After successful heparinization (ACT of 300 seconds), the ascending aorta was cannulated with an Embol-X cannula to collect debris, while the outflow graft was sewn into the ascending aorta in the standard fashion with a partial cross clamp. Opening of the inferior right pericardial edge to the inferior vena cava, with release of stay sutures, allowed cardiac positioning with the apex out of the chest as in an off-pump CABG procedure. Meanwhile, the patient was placed in the Trendelenburg position, slightly rotated towards the surgeon. An apical sewing cuff was sewn into the left ventricular apex by passing a large silk stitch into the center of the apex, while 2-0 pledgetted Ticron mattress sutures were placed in the apex and passed through the cuff. Three large Foley catheters were prepared and ready for use. The first was moistened and passed into the inflow elbow of the ventricular assist device, positioned so the tip exits the proximal end of the cannula. Next it was inflated with 10 to 15 ml of saline to plug the lumen of the cannula, while the proximal end of the Foley was clamped to stop back bleeding. The second Foley catheter was prepared similarly and used to help core the LV apex, making sure it was inflated and deflated while still providing occlusion of the lumen. The final Foley catheter was prepared similarly and used in case of rupture of one of the first two Foley balloons. The outflow graft was then attached to the LVAD and back bled to help de-air the pump. The apex was then cored with a coring knife that was passed through the Foley and silk stay suture. An incision was made into the LV apex and the Foley was placed into the cavity. The second Foley was inflated with 15 ml of saline and traction applied to bring the apex up and out of the chest cavity. The apex was cored without entering the Foley, and scissors were used to remove the remaining cuff. After coring the apex, the inflow elbow was connected by deflating and removing the balloon. To minimize ejection of blood out of the hole during balloon removal and inflow graft connection, the heart was arrested for 1 to 2 minutes with adenosine intravenous bolus (6 to 12 mg IV) or fibrillated using DC fibrillator (Cardiovascular Instrument Corp., Model 2039, Wakefield, MA, USA). A linear incision was made through the diaphragm, allowing the pump to sit in the preperitoneal space and then the diaphragm was closed around the inflow cannula, while the pump was back bled again from the aortic side for de-airing.

## Results

The patients' baseline characteristics were measured in the intensive care unit prior to coming to the operating room (Table 1). The average patient age was approximately 50 years old and about two-thirds were male. Dilated cardiomyopathy was the most common etiology of heart failure and the average ejection fraction was approximately 15%. Of the 29 cases scheduled for OP LVAD, 27 cases were successfully performed with 18 patients as BTT and 7 as destination therapy. In one of the failed cases, the surgeon entered the right atrium during resternotomy. The second was a congenital patient with a previous Mustard operation who did not tolerate partial occlusion of the systemic great vessel. Both patients had successful implants with CPB.

**Table 1:** Demographics, hemodynamic values, and perioperative homeostatic parameters for off-pump device implantation for 27 patients.

Demographics		
Age (years)	Mean 50	SD ± 11.9

Gender	Male 18, female 9	

Primary pathology	Dilated CM* 10, Ischemic heart disease (IHD) 9, Postpartum CM* 1, Familial CM* 1, Congenital 1, Unknown 1, Drug-induced CM* 4	

Indication of operation	DT† 19; BT‡ 8	

**Hemodynamic Variables**		

Cardiac output (liters/minute)	4	SD ± 1

Ejection fraction (%)	15	SD ± 6

LVEDD§ (cm)	7	SD ± 1

Mean pulmonary artery pressure (mmHg)	37	SD ± 7

Preoperative inotropic support	No 10; Yes 17	

**Preoperative Homeostatic Parameters**		

Hemoglobin (gm/dl)	11	SD ± 1

Hematocrit (%)	34	SD ± 4

Platelet count (×1000/ml)	205	SD ± 69

aPTT|| (seconds)	51	SD ± 24

INR¶	1	SD ± 0.5

*CM: Cardiomyopathy; †DT: Destination therapy; ‡BT: Bridge to transplant; §LVEDD: Left ventricular end diastolic diameter; || aPTT: activated partial thromboplastin time; ¶INR: International normalized ratio

Statistical analysis was performed on 27 patients. Of those analyzed, 4 patients presented with cardiogenic shock. No patients required unplanned right VAD placement. Subsequently, 5 patients had a cardiac transplantation. Of these 5 patients, 2 had a heart transplant 1 year after LVAD insertion; 1 patient was transplanted 2 months after the LVAD placement and died 8 months posttransplant from graft rejection; and the other 2 patients underwent heart transplants 2 and 5 months after LVAD implantation. We excluded those 3 patients who received transplants after less than one year when calculating the mortality. Two patients categorized as destination therapy became transplant eligible.

Eight of 27 patients experienced postoperative bleeding, which required a transfusion of more than 4 units of PRBCs for an overall incidence of 30%. One patient returned to the operating room for re-exploration due to excessive bleeding. Postoperative results are presented in Table 2. The mean of postoperative hospital stays was 28 days with a standard deviation of 19. The average ventilation hour was 66 with a standard deviation of 93. There was no incidence of infection reported. There was 1 incidence of stroke, 6 of renal failure, 8 of respiratory failure, and 4 reintubations out of 27 operations. Causes of death in one year included sepsis with multi-organ failure (n = 6), coagulopathy with bowel necrosis (n = 1), and respiratory distress with right heart failure (n = 1). The percent of survival was 93% at 30 days, 76% at 90 days, and 67% at one year. According to a recent study on LVAD [6], the estimates of one-year survival rates were 68% for the continuous-flow LVAD and 55% for the pulsatile-flow LVAD. Our study showed similar one-year survival rates with continuous-flow LVAD and was better than the pulsatile-flow LVAD.

**Table 2:** Postoperative outcome, complications, and mortality in 27 patients undergoing off-pump left ventricular assist device implantation.

Postoperative Outcome		
	**Mean**	**Standard Deviation**

**Ventilation Hours**	66	93

**Postoperative Hospital Stay (days)**	28	19

**Postoperative Complications**		

	**Number of Incidence/Total**	**Incidence Rate**

**Infection**	0/27	0%

**Stroke**	1/27	4%

**Renal Failure**	6/27	22%

**Respiratory Failure**	8/27	30%

**Reintubation**	4/27	15%

**Postoperative Mortality**		

**Mortality at 30 days**	2/27	7%

**Mortality at 90 days**	6/25	24%

**Mortality at 1 year**	8/24	33%

To investigate whether our OP LVAD technique improved over time, we compared the mortality rates of different implantation periods (Table 3). We divided the total study into three time intervals (2004-2005, 2006, and 2007). The death rate during this time decreased from 50% to 17%. Even though the trend was not statistically significant (p = 0.08, Fisher's exact test), we believe the limited sample size played a role. It will be promising and worthwhile to validate this trend in a large clinical trial and/or a multi-center study.

**Table 3:** Data in tertiles over three-year period.*

Year of OP LVAD	Number of deaths at 1 year postoperative/Total	Percent of death
2004-2005	3/6	50%

2006	4/12	33%

2007	1/6	17%

*The total study was divided into three tertiles (2004-2005, 2006, and 2007). There is a trend of decreased death from 50% to 17% over a one-year period, reflecting progressive improvement in the perioperative experience of OP LVAD insertion with reduced postoperative mortality rate. The trend is not statistically significant (p = 0.08 Fisher' exact test) due to the limited sample size.

## Discussion

This study reports the feasibility of a surgical technique for placement of LVADs through a median sternotomy incision without the use of CPB at our institution. This technique was used in the implantation of several different LVAD devices and was successful in 27 of 29 consecutive patients. Our initial anesthetic learning experience with OP LVAD insertion was satisfactory. Our one-year survival of OP LVAD insertion was 67%, which proved better than the Randomized Evaluation of Mechanical Assistance for the Treatment of Congestive Heart Failure (REMATCH) trial (52%) and the post-REMATCH era (56%). We believe this may be related to the avoidance of CPB, although this hypothesis has yet to be proven [2,4]. This was also comparable to the one-year survival of the most recent large trial using continuous-flow LVAD (68%), and better than pulsatile flow LVAD (52%), both on CPB [7]. However, our results were slightly less favorable compared with the one-year survival (73%) seen in recent trials conducted by Pagani et. al, using continuous-flow rotary LVAD using CPB in BTT patients, which correlated consistent improvements in patient outcome with newer device technology [8]. However, we believe that avoidance of CPB combined with improvements in device technology could provide greater benefits in future clinical outcomes.

In our series of 27 OP LVAD insertions, 9 patients experienced postoperative bleeding leading to transfusion of more than 4 units of PRBCs in the first 24 hours, a rate better than the historical REMATCH trial group (38%) in which all procedures were performed using CPB. In another study, using the same definition for massive perioperative bleeding as the INTERMACS registry and our study, which evaluated 222 patients with a Novacor LVAD implanted during CPB, massive perioperative bleeding occurred in 97 out of 222 patients and half of the total population returned for reoperation [9,10]. We believe this may be related to CPB and its deleterious effect and/or the Novacor device. However, this claim warrants further investigation in a randomized control trial with newer technology like the HeartMate II LVAD, which has different characteristics of flow.

Despite our initial encouraging results, this study has a number of limitations. While it does represent a consecutive series of patients who underwent the same surgical technique, it is a retrospective study that consists of a limited number of subjects and the hypothesis was generated after the surgical technique was established. In addition, due to the retrospective nature of the data collection, the transfusion trigger was not standardized within the first 24 hours postoperatively. Lastly, we could not do a case match control group from historical data in our institution for two reasons: 1) there was an upgrade in device technology, and 2) all of these cases were performed by one surgeon, who was using only this technique in 27 consecutive patients, and the historical group cases were performed by different surgeons on the CPB.

## Conclusions

As we performed more procedures off pump, the perioperative team became more familiar with the surgical technique and anesthetic requirement, and this was reflected in our data, showing improvement over time in one-year survival post implantation. The death rate over one year decreased from 50% to 17% in our small series, although this trend was not statistically significant due to limitations in sample size; however, many events including postoperative care, etc. can affect the one year outcome. It did show promise in helping high-risk heart failure patients, and the next step is to perform this technique in a larger clinical trial to validate this hypothesis, which we believe is worthwhile in improving clinical outcomes of cases done off CPB. This would allow confirmation of our findings, while addressing the limitations of our retrospective analysis. In conclusion, based upon our findings, OP LVAD implantation is a feasible surgical technique, and combining this technique with improved device technology could provide even greater improvement in patient outcomes in the future.

## Competing interests

The authors declare that they have no competing interests.

## Authors' contributions

All authors have read and approved the final manuscript. HA: Designed study, analyzed data, and wrote manuscript. MA: Collected and analyzed data. JH: Analyzed data and wrote manuscript. GD: Provided anesthesia for cases, analyzed data, and wrote manuscript. LY: Provided statistics. BS: Invented technique and surgeon on cases.
